# Africa and zero hunger agenda: genome editing policy landscape, challenges and opportunities

**DOI:** 10.3389/fbioe.2025.1526851

**Published:** 2025-03-12

**Authors:** Olalekan Akinbo, Bongani Nkhabindze, Josephine Amedu, Rufus Ebegba, Agnes Asagbra, Billy Omboki Ratemo, Susan Angira Dada, Anne Muia, Roy Mugiira, Lilian Chimphepo, Leeford Oko Wristberg, Mathurin W. Rouamba, Koussao Some, Valter N. A. Nuaila, Alsácia Atanásio, Olufunke Bolatito Shittu, Charles Oluwaseun Adetunji, Loubna Hamidi, Elinasi Monga, Silas Obukosia, Muyiwa Seyi Adegbaju, Samuel Acheampong, Estherine Fotabong

**Affiliations:** ^1^ African Union Development Agency – NEPAD (AUDA-NEPAD), Directorate of Agriculture, Food Security and Environmental Sustainability, Midrand, South Africa; ^2^ National Biosafety Management Agency, Abuja, Nigeria; ^3^ National Biosafety Authority, Nairobi, Kenya; ^4^ National Biosafety Authority, Lilongwe, Malawi; ^5^ National Biosafety Authority, Accra, Ghana; ^6^ Agence Nationale de Biosécurité (ANB), Ouagadougou, Burkina Faso; ^7^ Centro Nacional de Biotecnologia e Biociencias (CNBB), Maputo, Mozambique; ^8^ Department of Microbiology, College of Bioscience, Federal University of Agriculture, Abeokuta, Nigeria; ^9^ Department of Microbiology, Edo State University, Auchi, Nigeria; ^10^ International Union for Conservation of Nature (IUCN), Dar es Salaam, Tanzania; ^11^ Department of Biomedical Sciences, College of Health Sciences and Technology, Rochester Institute of Technology, Rochester, NY, United States; ^12^ Department of Molecular Biology and Biotechnology, University of Cape Coast, Cape Coast, Ghana

**Keywords:** food security, genome editing, technophobia, policy landscape, agriculture biotechnology

## Abstract

Africa has historically struggled to adopt innovative agricultural technologies, which has significantly hindered efforts to ensure food security and improve livelihoods over the past century. A major obstacle in this regard has been the persistent skepticism surrounding the potential benefits of agricultural biotechnology. The challenges contributing to this skepticism include a notable knowledge gap among stakeholders, widespread technophobia, or fear of technology, as well as inconsistencies with global agreements such as the Convention on Biological Diversity (CBB). Although these challenges are not exclusive to Africa, they disproportionately impact the continent, making the need for effective solutions even more urgent. This paper investigates the national government policy landscape in five African countries that are poised to create a regulatory environment conducive to deploying genome editing technology for improved agricultural productivity. This exploration aligns with the continental agricultural policy initiatives, notably the “CAADP Malabo Declaration” and the soon-to-be-signed “CAADP Kampala Declaration.” Aligning with the African Union’s continental agenda on agricultural transformation, as outlined in the Malabo Declaration and other key documents, is crucial for adopting innovative agricultural technologies like genome editing. Such alignment becomes increasingly critical for realizing the objectives set forth in the post-Malabo Declaration, with the Kampala Declaration playing a vital role in its implementation. This cohesive approach will not only foster agricultural innovation but also expedite development across the continent, addressing the pressing needs of food security and livelihoods in Africa.

## 1 Introduction

Global society and governments face the significant challenge of feeding over 7 billion people. In response, the United Nations established Agenda 2030 on Sustainable Development Goals (SDGs) to achieve peace and prosperity worldwide. Among the goals, Sustainable Development Goal 2 aims for zero hunger by ensuring food security and nutrition through sustainable agriculture. The magnitude of this issue is underscored by alarming statistics: in 2019, approximately 8.9% (690 million) of the global population suffered from hunger, while about 2 billion people were deprived of safe, nutritious, and adequate food. Notably, over 50% of these individuals reside in Asia, with 205 million in Latin America and a striking 675 million in Africa.

The Food and Agriculture Organization (FAO) defines “hunger” as “an uncomfortable or painful physical sensation caused by insufficient consumption of dietary energy.” This sensation becomes chronic when an individual consistently fails to consume enough calories to maintain a normal, active, and healthy life. Hunger is particularly prevalent on the African continent, where the prevalence of undernourishment (POU) was about 192.6 million in 2005—this number increased to 250 million by 2019. Projections suggest that the POU could reach 433 million by 2030.

Climate change significantly exacerbates food insecurity globally, impacting approximately 700 million people who face food insecurity due to climate-related factors, as noted by IFAD and WHO. (2020). Rising temperatures associated with climate change reduce water availability for agricultural use, while land scarcity driven by urbanization, population growth, and desertification further constrains agricultural production. Specifically, the effects of increasing temperatures intensify water scarcity by increasing evaporation from water sources, altering precipitation patterns, and accelerating the melting of glaciers and snowpack, all of which disrupt the reliability of water supplies for agricultural activities. Concurrently, the drivers of land scarcity uniquely affect agricultural viability: urbanization transforms arable land into urban areas, population growth amplifies competition for the remaining land resources, and desertification degrades soil quality due to unsustainable land-use practices (Al-Mulali and Che Sab, 2018; Sulemana and Machol, 2023).

Given the critical role that agriculture plays in many developing economies, particularly in Africa—where it contributes around 14% to GDP and employs nearly 53% of the workforce—finding sustainable solutions is essential (Painter et al., 2022). Estimates indicate that sustainable agricultural practices must facilitate a 70% increase in global food production by 2050 to meet the demands of a population projected to reach nine billion people (Khandelwal et al., 2022; Sulemana and Machol, 2023). Thus, effectively addressing the challenges posed by climate change is vital for enhancing agricultural productivity and ensuring food security for the growing global population.

The continuous improvement in agricultural production has played a significant role in shaping human history. As the global population continues to grow, the adoption of sustainable agrifood systems is crucial for ensuring food security and achieving Sustainable Development Goal 2 (SDG 2). To achieve this, key improvements in the agricultural sector are necessary, including transitioning to more productive environments, enhancing agricultural management practices, selecting superior seeds or breeds, and adopting innovative production techniques that leverage genetic advancements (FAO, 2022). Recent insights into molecular genetics have further accelerated crop and animal improvements, significantly amplifying production capabilities. Among the innovative agricultural tools emerging today, genome editing (GEd) stands out due to its ability to enhance plant and animal genetics in ways that directly improve food security. When compared to traditional breeding methods, genome editing is more efficient and precise, significantly reducing the time needed to develop new crop varieties or animal breeds while allowing for targeted alterations in genetic make-up. The application of GEd can enhance the resistance of crops and animals to pests and diseases, improve nutritional content, shorten production cycles, and bolster resilience to abiotic stresses induced by climate change, thus exemplifying the potential of advanced agricultural technologies in supporting global food security.

However, these new agricultural innovations, notably involving genome editing and genetically modified organisms (GMOs), are subject to both international and domestic regulations. Member States of the African Union are encouraged to create regulatory frameworks governing these new genomic technologies and their applications. Such regulations must ensure the safe and sustainable utilization of innovations aimed at enhancing food security. Currently, the development, implementation, and enforcement of these regulatory frameworks vary significantly across countries, with some nations having more advanced policies, legislations, and guidelines in place than others (Masehela and Barros, 2023). Hence, the aim of this review is to explore the genome editing policy landscape in Africa, focusing on key countries such as Kenya, Nigeria, Malawi, Ghana, Mozambique, and Burkina Faso. These nations are not only developing but are also finalizing guidelines for the application of emerging genomic technologies. By examining the regulatory frameworks in these countries, this paper highlights how flexible and adaptive policies can encourage bio-innovation while simultaneously addressing socio-economic and environmental challenges.

## 2 Context and background

### 2.1 Agricultural biotechnology in Africa

Empirical evidence unequivocally demonstrates that agricultural innovations are essential in addressing the complex challenges that hinder food security. In this context, Africa has made significant strides in adopting agricultural biotechnologies to enhance crop production. The continent has utilized various plant biotechnology tools, including traditional methods such as hybrid technology, plant tissue culture, micropropagation, and molecular marker-assisted breeding (Akinbo, 2008; Akinbo et al., 2007; Akinbo et al., 2010). One notable example is the use of hybrid technology to improve agricultural production in Africa, which dates back to the early 1980s. Egypt pioneered local hybrid rice varieties in collaboration with the International Rice Research Institute (IRRI) (El-Mowafi et al., 2009; El-Mowafi et al., 2019). This initiative led to the development of 50 local hybrids by the African Rice Center by 2000, culminating in the commercialization of ISRIZ09 in Senegal in 2017. The African Agriculture and Technology Foundation (AATF) has also played a crucial role in developing and testing two-line rice hybrids across the continent, partnering with IRRI through the Alliance for Hybrid Rice in Africa to enhance adoption and capacity building (El-Namaky and Demont, 2013; Abebrese and Yeboah, 2020). In addition to rice, other crops have benefited from hybrid technology. For instance, South Africa introduced hybrid maize in 1949 (Fischer, 2022), leading to extensive collaboration among organizations such as CIMMYT and AATF, as well as national agricultural research institutions and seed companies, to develop and distribute hybrid maize effectively to farmers.

While maize hybrids have gained significant traction due to their staple crop status, both public and private sectors have also produced hybrid varieties of diverse crops, including lettuce, broccoli, eggplant, strawberries, and citrus fruits, which demonstrate increased resilience to environmental stresses. In parallel with hybrid technology, other biotechnology tools have emerged as essential components of Africa’s agricultural innovation landscape. Plant tissue culture, for example, has facilitated the growth and dissemination of disease-free plant materials, enhanced the production of secondary metabolites, and allowed for the cultivation of various crop species in bioreactors (Brink et al., 1998; Moyo et al., 2011). Furthermore, molecular marker breeding tools have revolutionized the development of new plant varieties, reducing the commercialization timeline from an average of 10–25 years to merely 7–10 years (Akinbo et al., 2012; Okogbenin et al., 2012). The availability of publicly accessible plant genome sequences has fostered the successful application of this technology, which is grounded in the understanding that an organism’s DNA encodes its phenotypes. Across Africa, researchers have employed molecular markers to tackle challenges such as resistance to bacterial blight, enhanced vitamin content, and submergence tolerance in crops (Verdier et al., 2012; Oladosu et al., 2020; Datta et al., 2021). Additionally, these markers have proved invaluable for screening progeny for diversity, developing resistant and tolerant varieties, and creating germplasm for research purposes, thus improving the efficiency and effectiveness of plant breeding programs across Africa (Olasanmi et al., 2021; Conde et al., 2024). As Africa continues to navigate the complex challenges of food security, there are substantial opportunities to enhance resilience through innovative solutions, particularly as genetic engineering and genome editing technologies gain momentum in the region.

### 2.2 Genetic engineering

Forty-eight countries in Africa have signed the Cartagena Protocol on Biosafety (CPB), reflecting a significant commitment to enhancing biosafety regulations. However, there’s a notable disparity between commitment and implementation, as only about a dozen countries have functional biosafety systems in place, with Rwanda being the latest to join the ranks (Mackenzie et al., 2003; Timpo, 2019; Nduwumuremyi, 2024). This gap underscores the need for sustained support and development of biosafety infrastructure across the continent. In terms of genetically modified (GM) crop commercialization, South Africa is leading the charge, with a substantial number of approved GM crops and acreage under cultivation. Other countries, including Kenya, Nigeria, and Ghana, have also begun to approve various GM crops for commercial production, indicating a growing acceptance of this technology (Gbadegesin et al., 2022).

To tackle the challenges associated with the adoption of GM crops, several organizations, including AUDA-NEPAD and ISAAA, are actively working to increase the number of functional biosafety systems. Their initiatives focus on harmonizing regulations governing GM trade and research while ensuring the protection of human, animal, and environmental health. As a result, these efforts aim to facilitate the responsible adoption of GMOs, which is gaining momentum across Africa. Key stakeholders such as the AUDA-NEPAD African Biosafety Network of Expertise (AUDA-NEPAD ABNE), the African Agriculture Technology Foundation (AATF), and National Agricultural Research Organizations (NAROs) have forged successful collaborations aimed at addressing the pressing challenges faced by small-scale farmers. These challenges include issues related to low yields, environmental safety concerns, and the need for skills transfer to utilize innovative products effectively (Gbadegesin et al., 2022; ABNE, 2017). Through these initiatives, the collaborative efforts of these organizations contribute to a more sustainable and productive agricultural sector in Africa, supporting technology uptake through training and capacity-building programs. By fostering an environment conducive to the responsible adoption of GM crops, these endeavors are poised to enhance food security and agricultural resilience across the continent.

### 2.3 Genome editing

The fourth platform making a significant impact on African agriculture consists of genome editing tools. As the continent grapples with the pressing need to enhance productivity and sustainably to ensure food and nutritional security, it recognizes the increasing importance of technology in agriculture. This acknowledgment has led to substantial strides in the adoption of genome editing techniques, effectively addressing the challenges posed by climate change and other emerging issues. Among these innovative methods, the CRISPR-Cas9 tool stands out for its extensive application in improving staple food crops. For instance, ongoing improvements are being made in crops such as bananas, with efforts dedicated to developing resistance and enhancing yield against diseases like Xanthomonas Campestris. Additionally, breeding initiatives for maize are actively targeting resistance to Maize Lethal Necrosis (MLN), which is currently undergoing national performance trials, and sorghum is being bred to resist the Striga parasite. Furthermore, researchers are working on developing herbicide-resistant yam varieties (Akinbo et al., 2021; Kaur et al., 2018; Boddupalli et al., 2020; Tripathi et al., 2022; Adegbaju et al., 2024).

Aligning with this technological shift, the African Union has recognized, in its Agenda 2063, the necessity of adopting and deploying emerging technologies like genome editing to transform the agriculture and food systems sector while simultaneously driving economic development forward. To facilitate this transformation, advisory opinions have been provided to member states through the African Union Panel on Emerging Technologies (APET) (African Union High-Level Panel on Emerging Technologies (APET), 2016). Consequently, countries such as Kenya, Nigeria, Ghana, and Malawi have utilized this advisory to develop science-based policies, creating robust frameworks for the regulation of research and trade in genome-edited products (Runo et al., 2024). The Cartagena Protocol on Biosafety (CPB) (Mackenzie et al., 2003) serves as a guiding global framework under which genome editing guidelines are established within national biosafety frameworks. These four countries undertook the development of their frameworks through broad stakeholder consultation forums, ensuring inclusive participation. Regulatory categorization hinges on whether novel (exogenous) DNA sequences are introduced into an edited product or not. For example, genome-edited organisms developed using the so-called site-directed nuclease (SDN)-1 and -2 mechanisms, which respectively use no-homologous end-joining (NEJ) and homology-directed repair sequences without inserts, are classified as conventional varieties when confirmation is provided that no novel DNA sequences were inserted into the resulting organism. Without such proof, these organisms will be categorized as GMOs. Conversely, products generated using the SDN-3 mechanism are unequivocally GMOs, as this approach, per definition, uses homology-directed repair sequences that include novel sequences/inserts, which are transferred to the edited host organism (Runo et al., 2024). To further promote the benefits of genome editing, AUDA-NEPAD spearheads communication and advocacy activities across eight countries: Kenya, Nigeria, Ghana, Burkina Faso, Mozambique, Malawi, Ethiopia, and Zimbabwe. These initiatives aim to raise awareness of the transformative potential of genome editing tools in achieving sustainable agriculture. The significance of these communications and advocacy efforts cannot be overstated; they serve as critical linchpins, translating scientific innovations into actionable solutions vital for realizing sustainable development not only in agriculture but across other sectors of the economy as well.

### 2.4 Biodiversity

Agriculture is a vital sector in Africa, contributing significantly to many countries’ GDP. To combat climate challenges, African nations have developed adaptive agricultural policies that incorporate biotechnology, alongside partnerships at international and regional levels for over two decades (Rock and Schurman, 2020). As climate change intensifies, prioritizing ecosystem health becomes crucial for human wellbeing, as the sustainability of agriculture is directly linked to the health of our ecosystems (He et al., 2021). Biotechnology and biodiversity play critical roles in addressing pressing global challenges while unlocking new economic opportunities. The conservation of biological diversity is essential for tackling issues such as population growth, soil degradation, and resource depletion (Tripathi et al., 2021). Through innovative applications, biotechnology has not only produced food and medicine but has also provided methods to regulate and enhance efforts aimed at combating biodiversity loss (Olomola et al., 2024).

The Convention on Biological Diversity, enacted in 1992, highlights the global commitment to conserving biodiversity, sustainably using biological resources, and equitably sharing benefits obtained from these resources (IUCN, 1994). Nonetheless, biodiversity loss remains a significant concern, with approximately one-third of plant species currently under threat. The biological diversity of ecosystems serves as the foundation for essential services, including water provision, food supply, and climate regulation. In light of this, many African countries recognize the severe importance of biodiversity and ecosystem services in promoting economic growth, sustainable development, and human welfare (AUDA-NEPAD APET, 2022). For instance, the unique marine ecosystems of the Western Indian Ocean provide local communities with food security, livelihoods, and natural beauty. This emphasizes the urgent need for sustainable management practices to protect these invaluable resources (IUCN, 2021).

However, despite the significance of biodiversity, human activities and climate change have led to substantial biodiversity loss across the continent. Healthy biodiversity and ecological productivity are essential for sustaining local economies and supporting communities (Worm and Lotze, 2021). Furthermore, the adoption of agricultural biotechnology in Africa has been sluggish, hindered by various complex socio-political, regulatory, and business factors (Wise, 2021; Neale et al., 2022). As climate change continues to present challenges, biotechnology emerges as a powerful ally in enhancing biodiversity and maintaining healthy ecosystems. By leveraging innovative biotechnological solutions, African nations can better navigate the complexities of agricultural sustainability and work toward a more sustainable future.

### 2.5 Challenges: knowledge gaps, technophobia, and inconsistencies with global agreements

Africa has made significant strides in creating an enabling environment for the adoption and deployment of agricultural biotechnologies, which are crucial for transitioning the agriculture and food systems sector toward sustainable pathways. However, several challenges continue to hinder the realization of maximum benefits from these emerging technologies. Foremost among these challenges is public perception, which is often shaped by concerns related to food and nutrition security, population growth, and environmental degradation. The existing knowledge gaps between developers, farmers, and the general public contribute to anxieties and misconceptions that impede the broader adoption of innovative agricultural practices. To tackle these issues, organizations such as AUDA-NEPAD, AATF, CGIAR, and the FAO emphasize the importance of stakeholder engagement. By fostering a common understanding among all parties involved and obtaining social licenses for biotechnology initiatives, these organizations aim to build trust and transparency. In parallel, it is essential that regulatory frameworks align with global agreements, such as the Cartagena Protocol on Biosafety. While many African countries have signed the protocol, they often struggle with its implementation, which further complicates the landscape for agricultural biotechnology.

In addition to these perception-related challenges, African agriculture faces dwindling public investments in research and development (R&D), with funding averaging only 0.42% of GDP. This is significantly lower than the global average of 1.7% (Adepoju, 2022), which limits the continent’s capacity to adequately respond to emerging agricultural challenges. Compounding this issue is weak farmer demand, which is largely a result of subsistence farming models. Addressing this requires targeted communication and advocacy strategies tailored to the needs of farmers and their communities. Furthermore, intellectual property (IP) ownership concerns can hinder the uptake of biotechnology. However, organizations like CGIAR and AATF are bridging this gap by accessing essential tools for the public good. While these initiatives support research and farmer adoption, more work is needed to address the complexities surrounding IP rights and ensure equitable access to biotechnology innovations.

Ultimately, a multi-faceted approach is necessary to overcome these challenges. This approach should encompass robust stakeholder engagement, regulatory harmonization, increased investment in R&D, and strategic communication designed to enhance demand for agricultural innovations. By fostering such an environment, Africa can pave the way for sustainable agricultural development, harnessing the full potential of biotechnologies to ensure food security and resilience against future challenges.

### 2.6 Continental policies: introduction to CAADP Malabo declaration and CAADP Kampala declaration

The Comprehensive African Agriculture Development Programme (CAADP) was established in 2003 when Heads of State and Government convened in Maputo and signed the Maputo Declaration. This pivotal agreement aimed to enhance agricultural productivity and food security across the African continent, rooted in the recognition that agriculture serves as the backbone of many African economies and is a primary source of livelihood for the majority of its people. To realize this ambitious goal, member states committed to allocating a minimum of 10% of their national income to agriculture, with the objective of achieving at least 6% annual productivity growth (CAADP, 2003).

In a further demonstration of commitment, the goals and targets outlined in the Maputo Declaration were reaffirmed a decade later during the 23rd session of the African Union (AU) assembly in Malabo, Equatorial Guinea. This session not only marked the 10-year anniversary of CAADP but also saw heads of member states reiterate their dedication to the principles and values of the programme. Key commitments made included the urgent aim of ending hunger by 2025, halving poverty through agricultural transformation, enhancing financial investments in agriculture, boosting trade in agricultural commodities among member countries, and improving the resilience of livelihoods against climate-related risks. Additionally, member states underscored the importance of mutual accountability regarding CAADP actions and results, alongside strengthening the AU to effectively support these commitments (CAADP, 2003).

As part of the latest CAADP biennial review, 49 member states submitted their reports to the commission in coordination with Regional Economic Communities (RECs). In this review, the benchmark for a country deemed to be on track rose from an initial score of 3.94 in 2017 to 9.29 for the 2023 evaluation. Alarmingly, however, no country currently meets the commitments set for 2025. This situation highlights the pressing need to accelerate the implementation of initiatives designed to build resilient agricultural systems in line with CAADP’s vision. Notably, the overall Africa-wide score for this biennial review stands at 4.56, reflecting an improvement from 4.32 in 2021 and 4.03 in 2019 (African Union Commission, 2024).

These recent developments have catalyzed the urgency to formulate a post-Malabo strategy and action plan for CAADP spanning the next decade (2025–2036). This strategy was reviewed and validated during a meeting in Kampala in August 2024. Notably, this progression aligns seamlessly with the African Common Position established at the UN Food Systems Summit in Ethiopia in February 2024. The Summit called upon the African Union Commission to develop a comprehensive strategy and action plan for the period from 2026 to 2035, which will be deliberated upon by heads of state and anticipated to culminate in the Kampala Declaration set for adoption in January 2025 (African Union, 2024).

## 3 Selected national policy and regulatory frameworks for genome editing

### 3.1 Kenya

After signing and ratifying the Cartagena Protocol on Biosafety (CPB) in 2003, Kenya made significant strides in biotechnology by developing and publishing the National Biotechnology Development Policy in 2006. This policy acknowledged the essential role of biotechnology in reducing poverty, improving food security, and conserving biodiversity and the environment. To ensure responsible advancement in the field, the Kenyan government created a biotechnology and biosafety policy, which serves as a strategic guide for the growth and application of biotechnology while safeguarding both citizens and the environment from potential harm. A key commitment of this policy was to conduct and manage risk assessments for all introduced GMOs. The policy also laid the groundwork for developing legislation that would regulate and protect the use of biotechnology products, all while fostering an environment conducive to the advancement and commercialization of biotechnology (National Biotechnology Development Policy, 2006).

In 2009, the Kenyan Parliament responded to the growing need for regulation by enacting the Biosafety Act, which specifically governs activities related to GMOs. This Act established the National Biosafety Authority (NBA, 2011) as the principal body responsible for coordinating and implementing biotechnology regulations within Kenya. With this legal framework, the NBA acquired the authority to oversee all actions involving GMOs across various sectors, including food, feed, research, industry, trade, and environmental release (Republic of Kenya, Biosafety Act, 2009).

The objectives of the Biosafety Act are multifaceted and aim to facilitate responsible research on GMOs while minimizing potential risks. Key goals include ensuring adequate protection for the safe transfer, handling, and use of GMOs that could impact human health and the environment, as well as establishing a transparent, science-based, and predictable process for reviewing and making decisions regarding various GMO-related activities (Republic of Kenya, Biosafety Act, 2009).

To enhance the regulation of GMOs at different stages of their development and use, four sets of regulations were established. The Biosafety Contained Use Regulations of 2011 guide activities involving GMOs in research settings such as laboratories and confined field trials (CFTs) (NBA-Kenya, 2011a). The Biosafety Environmental Release Regulations of 2011 cover the release of GMOs into the environment, their market placement, and commercialization (NBA-Kenya, 2011b). Additionally, the Biosafety Import, Export, and Transit Regulations of 2011 focus on ensuring the safe movement of GMOs into, within, and out of Kenya (NBA-Kenya, 2011c). Finally, the Biosafety Labeling Regulations of 2012 facilitate the labeling of approved GMO products for marketing (NBA-Kenya, 2012).

To date, the NBA has reviewed and granted approval for over 50 research projects and three environmental release applications. Notably, the environmental release approvals encompass Bt cotton (MON 15985), which is actively cultivated, Bt maize (MON 810), which is awaiting variety release, and virus-resistant cassava (VIRCA), currently undergoing national performance trials (NBA-Kenya, 2024). Unfortunately, these approvals have encountered resistance from various groups, which have led to litigation.

In light of recent advancements in genome editing technology, the NBA has developed guidelines expressly designed to determine the regulatory processes applicable to genome editing techniques and their resulting products. Recognizing that genome editing can produce organisms qualifying as GMOs or those indistinguishable from traditionally bred organisms, the Guidelines for Genome Editing in Kenya stipulate that genetic modifications may also create changes achievable through conventional breeding methods (NBA-Kenya, 2022).

**FIGURE 1:**
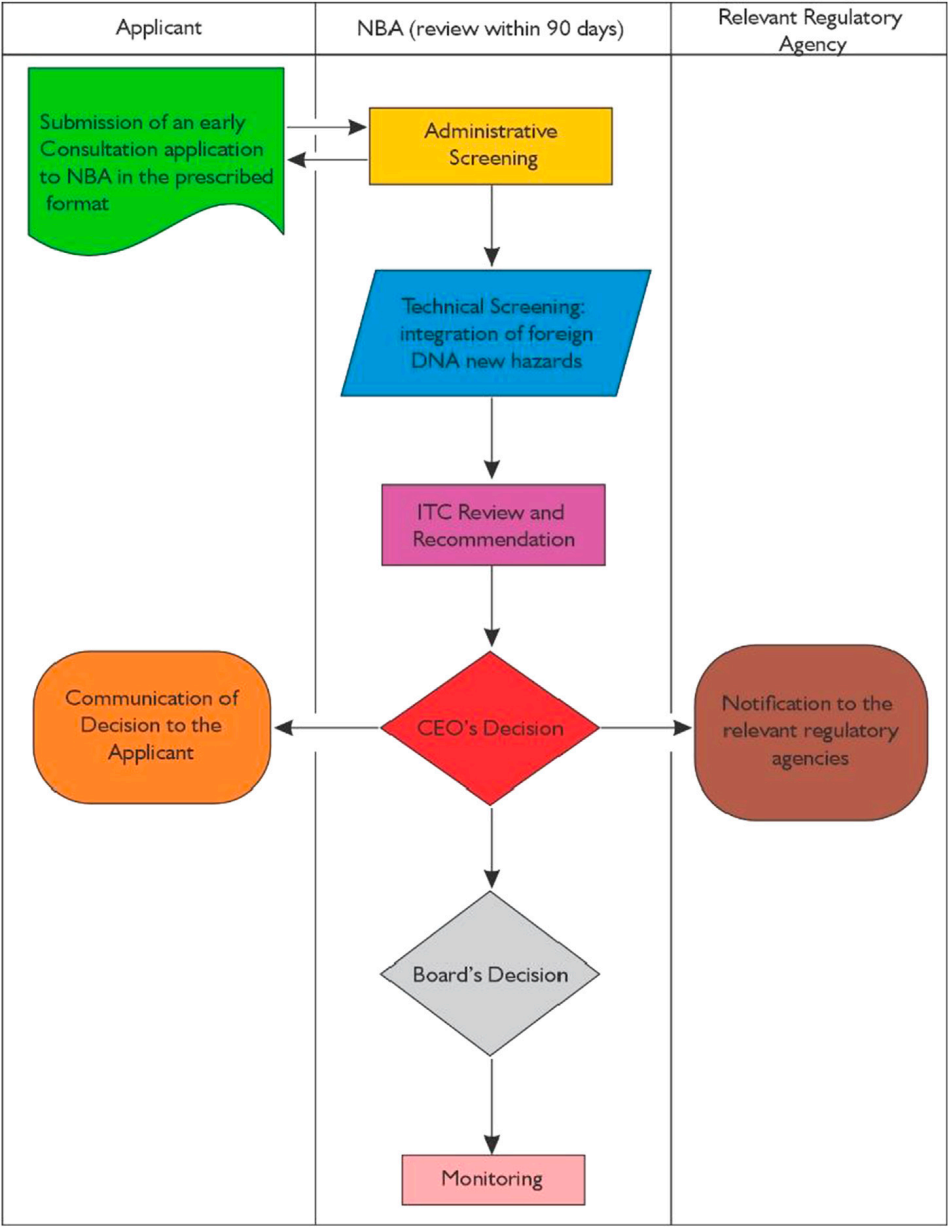
Flowchart for the Early Consultation on genome Editing process in Kenya.

According to the Biosafety Act of 2009, GMOs are classified as organisms possessing a novel combination of genetic material derived from modern biotechnology techniques (Republic of Kenya, Biosafety Act, 2009). This definition harmonizes with the Cartagena Protocol’s description of living modified organisms (Secretariat of the Convention on Biological Diversity, 2000). Consequently, it follows that any genome editing product that does not qualify as a GMO must be subjected to a different regulatory pathway (Figure 1). This distinction aligns with the Policy Framework for Applications of Genome Editing in African Agriculture, which advocates for a structured, harmonized, and scientifically sound regulatory system across the continent (AUDA-NEPAD APET, 2022). Such globally harmonized regulations could significantly enhance international trade in agricultural products while ensuring a high level of safety.

The Guidelines for Genome Editing in Kenya incorporate an expedited early consultation process, aligning closely with approaches adopted by numerous other countries (Figure 1). Within this framework, applicants are required to complete the designated Early Consultation Form and submit it to the NBA, supplying detailed information on the experimental procedures and resulting products. This process is applicable to all genome-edited plants, animals, and microorganisms involved in research, environmental release, market placement, import, export, and transit that the applicant seeks to classify as genome-edited organisms. The regulatory pathway is assessed on a case-by-case basis, depending on whether transgenic materials are involved (NBA-Kenya, 2022).

If a genome editing technique or derived product is classified as a GMO, the applicant is instructed to submit a GMO application in the prescribed format. Conversely, if a non-GMO determination is made, ongoing monitoring for unintended effects is mandated for the duration of the approval, adhering to biosafety requirements (NBA-Kenya, 2022).

This distinct regulatory pathway for genome editing not only conserves time and resources but also avoids the lengthy and costly GMO approval process. Currently, the focus has been on less commercially significant crops, such as striga-resistant sorghum, which is subject to field trials. Thus far, the NBA has reviewed seven early consultation applications for field trials (covering maize, sorghum, bacteria, and rice) that received non-GMO determinations. Consequently, regulation of these genome-edited products has transitioned to other relevant regulatory agencies, which are also partner entities of the NBA. Notably, the NBA has yet to receive any applications for the commercialization of genome-edited products.

### 3.2 Nigeria

Nigeria recognizes the significant potential of modern technologies to enhance food security, mitigate the impacts of climate change, and support economic growth for its rapidly expanding population. To harness these opportunities, Nigeria has implemented policies designed to facilitate research and the commercialization of biotechnology products (Adegbaju et al., 2024). Specifically, the National Biotechnology Policy, introduced in 2015, was established to oversee all activities related to modern biotechnology, including research, development, and commercialization efforts. Despite the acknowledged societal benefits of these biotechnologies, there is a consensus within the international community, including Nigeria, that a balanced and comprehensive approach to biosafety is vital. This approach ensures that effective measures are in place to manage any potential adverse impacts that these products may have on human health and the environment (Adewumi and Ilori, 2019).

From a regulatory perspective, Nigeria employs two key approaches to managing modern biotechnologies: leveraging existing legislation across diverse multi-sectoral agencies, and creating new biosafety laws with clear legal authority and centralized decision-making bodies (Tachikawa and Matsuo, 2023). Reflecting the common practice among African nations, Nigeria has adopted the latter approach. Furthermore, Nigeria is a signatory to the Convention on Biological Diversity (CBD) and the Cartagena Protocol on Biosafety (CPB), ratified in 1994 and 2003 respectively. These pivotal agreements laid the groundwork for developing a robust national regulatory framework aimed at addressing the potential adverse effects stemming from the transboundary movement, handling, use, and release of GMOs on human health, biodiversity, and the environment. In alignment with these multilateral agreements, the first and second National Biosafety Guidelines were established in 1994 and 2001, respectively, which culminated in the drafting of the National Biosafety Management Agency (NBMA) Bill that was subsequently enacted into law in 2015 (Adewumi and Ilori, 2019; Nwosu and Bello, 2023).

The NBMA (2015) established the National Biosafety Management Agency as the competent national authority overseeing all biosafety matters. The agency is tasked with providing a regulatory framework, along with institutional and administrative mechanisms to ensure safety measures are effectively implemented in the application of modern biotechnology in Nigeria. This framework aims to prevent any adverse effects on human health, biodiversity, and the environment (National Biosafety Management Agency Act, 2015). In 2019, the Act was amended to include provisions for regulating emerging biotechnologies, such as genome editing, gene drives, and synthetic biology, as well as measures to enhance biosecurity.

Under the NBMA Act of 2015 (as amended), Nigeria regulates several activities related to GMOs, which include contained use, confined/multi-locational field trials, commercialization, and the import and export of GMOs for food, feed, and processing purposes. With socio-economic and ethical considerations integrated into the regulatory process, the legislation emphasizes comprehensive oversight (Adewumi and Ilori, 2019; Nwosu and Bello, 2023). For activities involving contained use and confined field trials, responsible institutions are required to obtain accreditation and have their facilities certified by the NBMA. Additionally, research endeavors in modern biotechnology are supervised by Institutional Biosafety Committees (IBCs) within those institutions, which report directly to the Agency.

Regarding the release of GMOs into the environment—whether for confined or commercial use, or for food, feed, or processing—the NBMA Act mandates a risk assessment review process to be conducted on a case-by-case basis. This review must be transparent and scientifically rigorous, taking into account information provided by the applicant, public opinion, and scientific expertise from the Ad Hoc National Biosafety Committees and the National Biosafety Technical Sub-Committees. Furthermore, available data from reputable sources such as the Biosafety Clearing House (BCH), the Organization for Economic Co-operation and Development (OECD), and the European Food Safety Authority (EFSA) can also inform these assessments.

In a landmark achievement, Nigeria became the first African country to approve the commercialization of a locally developed GM food crop, the Pod-Borer Resistant Cowpea, in 2019. This crop expresses the Cry protein designed to combat the insect pod-borer, Maruca vitrata (Ehirim et al., 2020; Kedisso et al., 2022). Additionally, Nigeria has granted commercialization approvals for other crops, including Bt cotton, which is resistant to bollworms, and TELA maize, which tolerates moderate drought while also resisting the fall armyworm (Spodoptera frugiperda) and stem borers (Nwosu and Bello, 2023).

**FIGURE 2:**
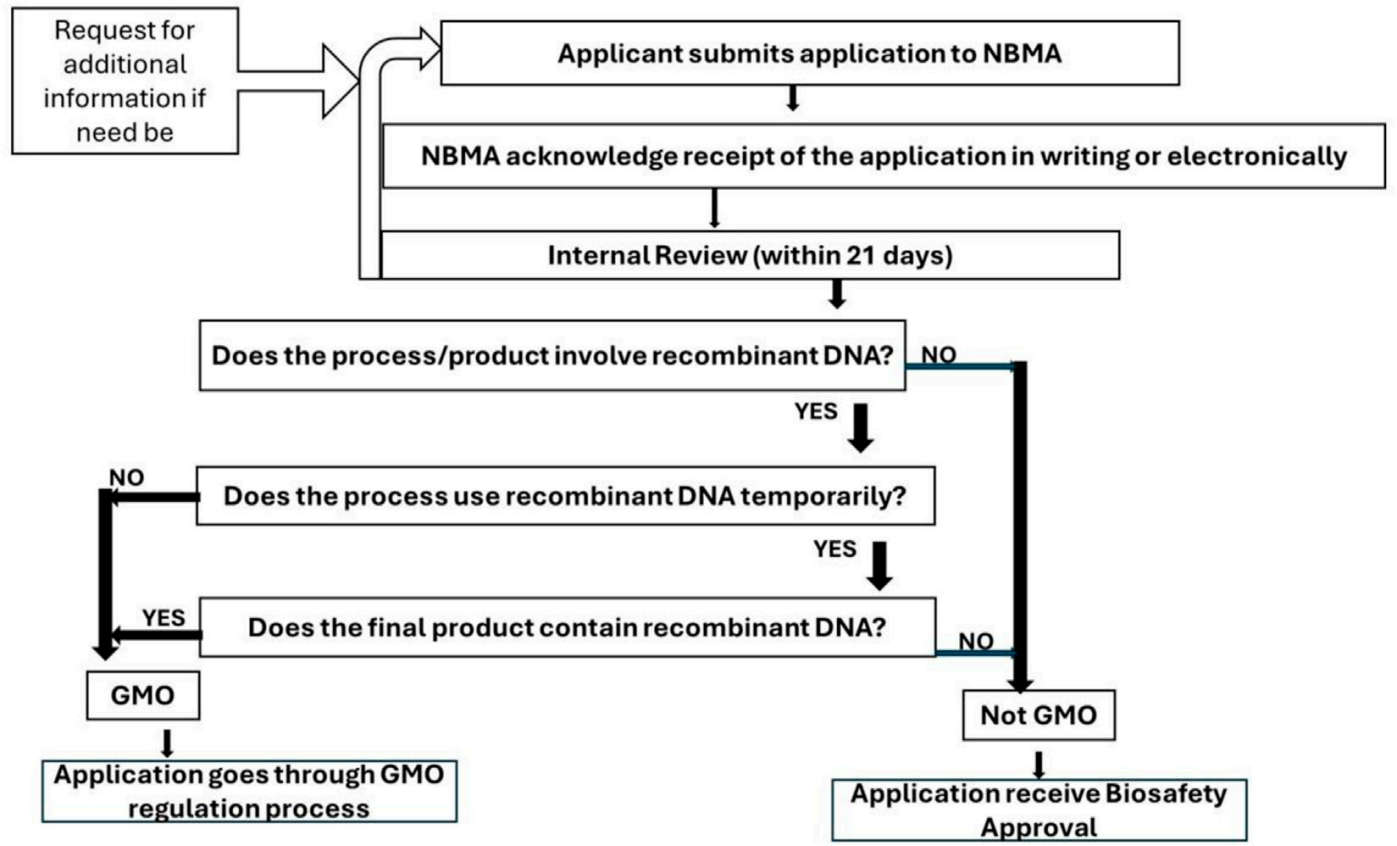
Flowchart for the Early Consultation on genome Editing process in Nigeria.

Moreover, in 2019, Nigeria positioned itself as one of the pioneer nations in Africa by amending its Biosafety Act to accommodate the regulation of emerging biotechnologies (Komen et al., 2020; Adegbaju et al., 2024). The National Guideline on Genome Editing was validated on 19 December 2020, outlining the necessary processes for all dealings related to genome editing within Nigeria, as well as the requirements for applications concerning genome-edited products. These guidelines stipulate that applicants engaging in genome editing or utilizing genome-edited products are required to apply, providing essential information to the National Biotechnology Authority (NBMA), which will ascertain the GMO classification of their product on a case-by-case basis. Despite the regulatory variances, all genome editing processes/products are subject to varying levels of regulatory oversight. If a genome editing process does not result in a new combination of genetic material, it falls under a non-GM regulatory classification that requires a Clearance Permit (Figure 2). Conversely, any process necessitating recombinant DNA or resulting in a new combination of genetic material will categorize the product as a GMO, thus placing it under GM regulations.

In summary, all dealings involving genome editing in Nigeria must secure approval from the NBMA, either through Clearance Permits or Approval Permits, contingent upon the regulatory classification. The agency determines the regulatory pathway based on an internal review following consultations with the applicant, thereby ensuring a level of accountability and scientific rigor in the management of biotechnology products.

### 3.3 Ghana

To fully harness the potential of genome editing products, a balanced approach is crucial, one that prioritizes safety, equity, and ethical considerations. Ghana is making strides in this direction, leveraging its well-established and comprehensive regulatory frameworks to advance genome editing technologies. These existing policies not only provide a solid foundation but also facilitate the effective adoption and integration of genome editing technologies within the country. By aligning safety and ethical standards with technological advancements, Ghana aims to ensure that the implementation of genome editing contributes positively to its agricultural and biotechnological landscape.

The Constitution of Ghana establishes a strong foundation for promoting science and technology through education. Specifically, Section 38(3) (a) of the 1992 Constitution mandates the state to ensure access to education at all levels, placing a particular emphasis on science and technology. This provision plays a crucial role in integrating genome editing into educational curricula, equipping students with the knowledge and skills necessary to advance in this rapidly evolving field. By prioritizing science and technology education, Ghana not only fosters a well-informed workforce but also enhances its capacity for innovation. This strategic focus ultimately contributes significantly to national development through the practical applications of genome editing, positioning the country as a leader in biotechnology advancements.

The Biosafety Act 2011 (Act 831) serves as the cornerstone of biotechnology regulation in Ghana, overseeing the safe development, transfer, handling, and use of genetically modified organisms and related technologies, including genome editing. Within this framework, the establishment of the National Biosafety Authority (NBA) plays a critical role in ensuring that genome editing activities are conducted in a safe and regulated environment. To further enhance this regulatory landscape, comprehensive guidelines were introduced in October 2023 under the Biosafety Act. These guidelines provide clear processes for risk assessment, management, and decision-making, effectively addressing legal and policy uncertainties through scientifically sound, case-by-case evaluations. They outline specific criteria to determine whether an organism or product derived from genome editing techniques falls under the regulation of the Biosafety Act. Notably, products resulting from genome editing that do not contain inserted genes or derivatives of foreign genes in the final product are exempt from regulatory control under the Act.

**FIGURE 3:**
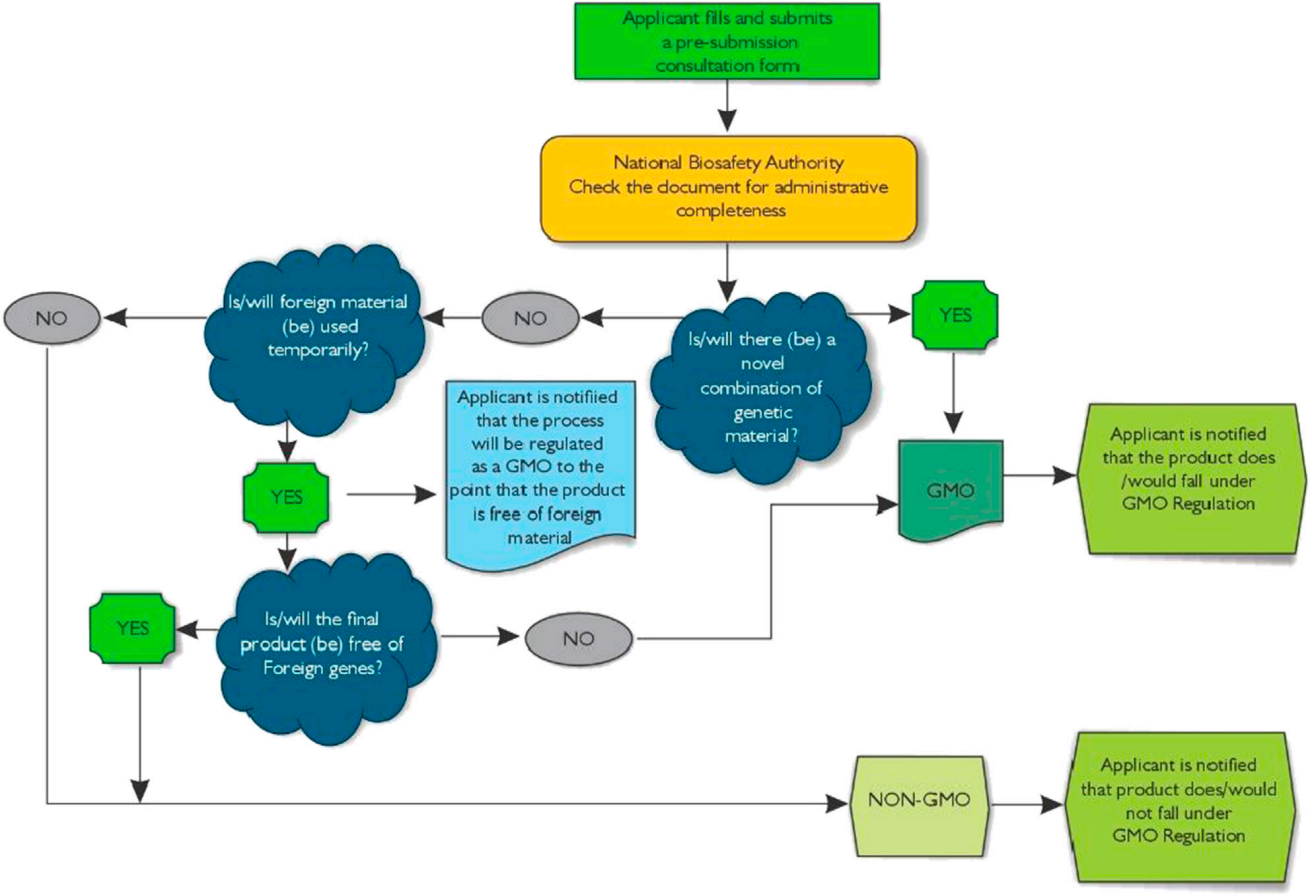
Flowchart for the Early Consultation on genome Editing process in Ghana.

In order to streamline the regulatory process, the NBA encourages applicants to participate in pre-submission consultations. This preliminary step involves completing a pre-submission consultation form, which helps ascertain the regulatory status of the application and provides a systematic approach to ensure predictability in decision-making (Figure 3). Such consultations equip applicants with a thorough understanding of the regulatory requirements and assist in aligning their submissions with the established guidelines, thereby potentially reducing delays in the approval process. Additionally, the regulatory focus centers on the classification of genome-edited products based on whether they contain inserted genes and the nature of these insertions. By employing a case-by-case approach to decision-making, the guidelines allow for flexible and adaptive regulatory measures tailored to the specific applications of genome editing and their potential impacts.

Crucially, the regulatory guidelines for genome editing are designed to be dynamic; they will undergo periodic review and updates in light of new scientific information. This iterative process ensures that the guidelines remain current and relevant, effectively reflecting the latest advancements in genome editing technologies and best practices in biosafety. Through the implementation of these comprehensive guidelines, Ghana significantly strengthens its capacity to manage genome editing technologies responsibly. This fosters an environment that is conducive to innovation while simultaneously ensuring safety and compliance with regulatory standards.

### 3.4 Burkina Faso

Burkina Faso established a regulatory framework for products derived from modern biotechnology in 2004, starting with a pivotal decree (No. 2004–262/PRES/PM/MECV/MAHRH/MS) adopted on 18 June 2004, which outlined safety protocols for biotechnological applications. In alignment with this decree, the government created the National Biosafety Agency (ANB) in 2005 as the competent national authority to oversee biosafety in the country. The government established the ANB alongside two advisory bodies, which play critical roles in the regulatory process. The first is the National Scientific Committee on Biosafety, tasked with assessing applications related to GMOs and conducting risk evaluations. The second body is the National Biosafety Observatory, responsible for public education, awareness, and monitoring to prevent potential harm. The initial framework facilitated Burkina Faso’s first authorization request in 2005 for confined environmental experimentation with Bt cotton (Bollgard II), designed to resist the insect pest *Helicoverpa armigera*. The regulatory evolution continued with the passage of an initial biosafety law in 2006. Lawmakers revised this law in 2012 to address compensation and liability aspects, following Ghana’s adoption of the Nagoya-Kuala Lumpur Supplementary Protocol in 2010.

From 2005 to the present day, the ANB has been actively involved in reviewing and granting authorizations for various biotechnological experiments, including contained and controlled environment studies and environmental releases. Key projects within this framework have focused on developing genetically modified crops, including herbicide-tolerant Bt cotton, insect-resistant maize, and cowpeas resistant to pod borers. Additionally, researchers have worked on genetically modified mosquitoes and rice with built-in resistance to bacterial blight. Scientists used CRISPR/Cas9 gene editing to create a new product. They removed and replaced specific genetic material that help produce sugar, which is essential for the growth of the Xanthomonas oryzae bacterium. The dossier for this novel rice variety underwent thorough analysis by the National Biosafety Agency, including risk assessments and management measure evaluations, in accordance with existing regulations, before receiving authorization for greenhouse experimentation for a duration of 2 years.

**FIGURE 4:**
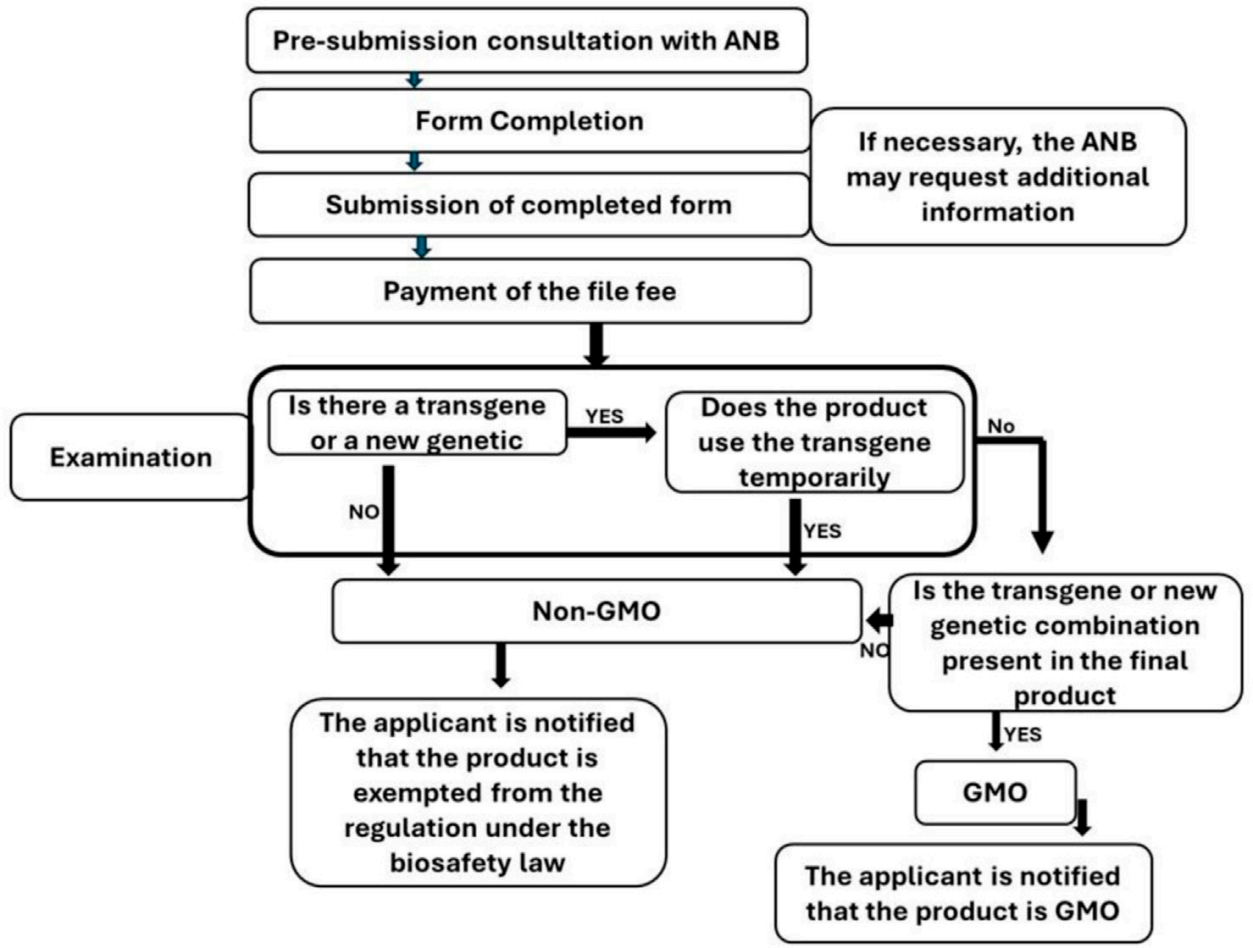
Flowchart for the Early Consultation on genome Editing process in Burkina Faso.

However, the review of the edited rice dossier underscored the necessity for adapting and revising the existing regulations in Burkina Faso. Notably, the absence of a transgene or a new genetic combination in the edited rice suggests a lower risk profile compared to conventional GMOs. Consequently, in 2023, the ANB initiated a comprehensive review of its regulatory practices and created a technical guide to modern biotechnology regulations, particularly focusing on genome editing (Figure 4). This guide delineates the types of organisms and products that are subject to regulation as well as those exempted by law and treated as conventional varieties or breeds. The ANB will exempt genetically modified organisms from regulation under Biosafety Law 064-2012/AN if they meet two conditions. First, the ANB must verify that the final product contains no foreign genes. Second, the organisms must not combine genes from incompatible species.

This adoption of the technical guide enabled the National Biosafety Agency to issue Decision No. 2023-000122/MESRI/SG/ANB/DG on 21 July 2024, authorizing the use of edited rice lines to combat bacterial blight in rice. After securing approval, the farm took a major step forward by testing the genetically edited rice during the 2023–2024 crop year, demonstrating the practical application of biotechnology in Burkina Faso’s agricultural sector.

### 3.5 Malawi

Malawi’s economy is predominantly agro-based, with agriculture contributing approximately 30% to the gross domestic product (GDP) and employing more than 80% of the population (NSO, 2019). Recognizing the essential role of innovations and technologies, including biotechnology, in enhancing agricultural productivity, the country seeks to fulfill its aspirations as outlined in the Malawi 2063 development plan. To achieve these goals, Malawi has established mechanisms to ensure that biotechnology is effectively regulated, prioritizing the safety of both the environment and human health. Central to this regulatory framework is the country’s comprehensive biosafety legal structure that governs biotechnology activities. Among these foundational regulations is the Biotechnology and Biosafety Policy of 2008, along with the Biosafety Act of 2002 and the Biosafety Regulations of 2007, which specifically address the management of GMOs. Between 2001 and 2002, genetically modified maize in food donations to Malawi triggered a food crisis, which in turn underscored the need for a robust biosafety framework. In response to this pressing issue, the government expedited the development of its legal framework to ensure the appropriate regulation of future GMOs entering the country.

**FIGURE 5:**
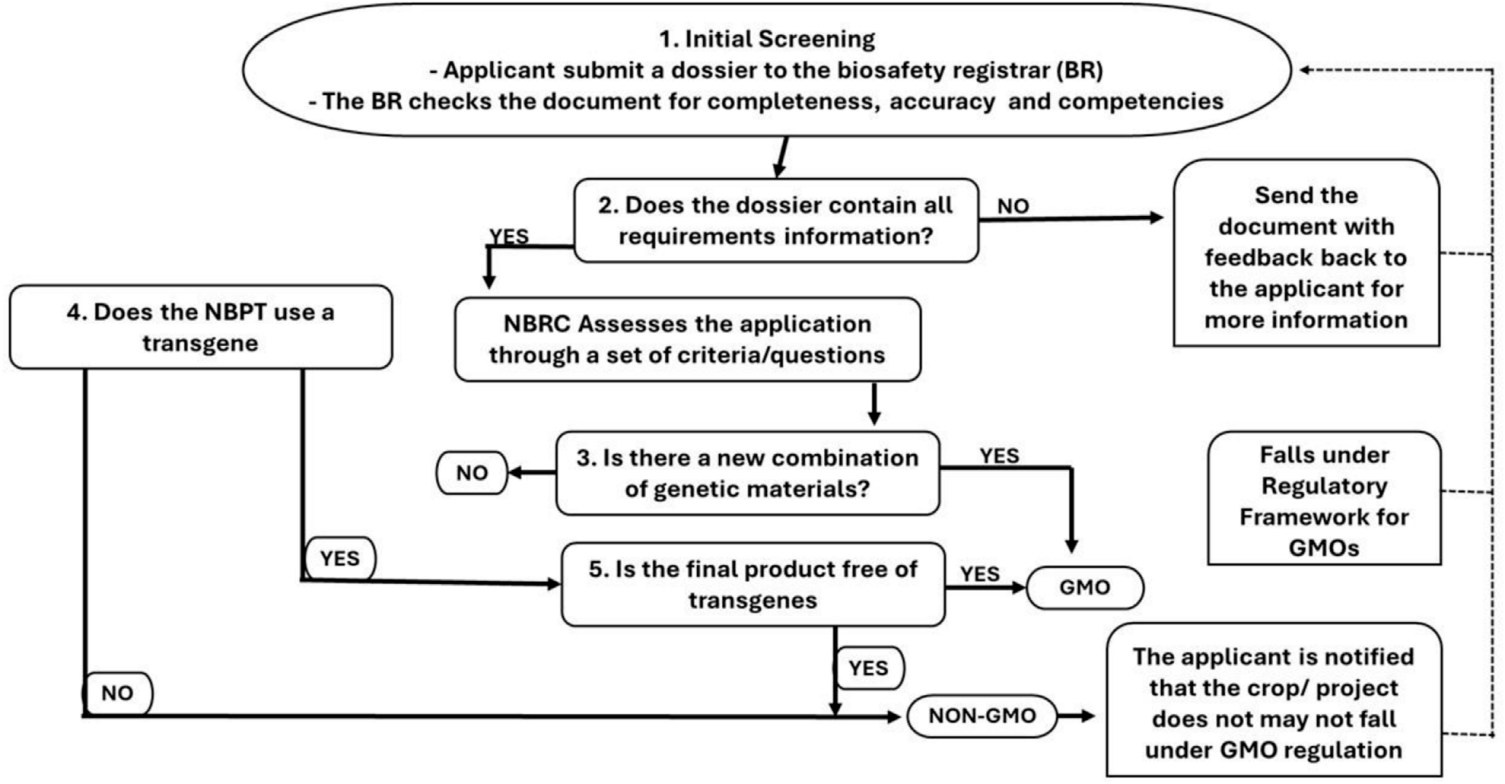
Flowchart for the Early Consultation on genome Editing process in Malawi.

Further demonstrating its commitment to biosafety, Malawi became a party to the Cartagena Protocol on Biosafety in 2009. Authorities developed guidance documents to complement this step, facilitating effective implementation of the Protocol and national biosafety laws. These documents include guidelines for conducting confined field trials and multi-location trials, guidelines for GMOs with stacked genes, guidelines for safety assessments for food and feed derived from genetically modified crops, as well as guidelines for determining the regulatory processes for genome-edited plants and their products. Although the current laws do not specifically address genome editing, these guidelines provide a framework for regulating genome-edited crops. The Genome Editing (GEd) Guidelines classify products resulting from genome editing as conventional if they do not contain recombinant DNA. In contrast, the guidelines regulate products with transgenes in the same way as other GMOs under the Biosafety law (Figure 5). Regulators thoroughly investigate product development to detect the use of transgenes and their presence in the final product, underscoring the product-based regulatory approach. A flowchart illustrates the steps followed in the regulation of genome-edited products, highlighting the thorough assessments mandated by the guidelines.

Building on this regulatory framework, the National Biosafety Regulatory Committee, established under the Biosafety Regulations of 2007, reviews all biotechnology applications, including those related to genome editing activities, and makes recommendations to the Minister responsible for the Environment for approval. Although Malawi has not yet received any applications for the development or importation of genome-edited products, the country is taking proactive steps to promote genome editing. Two experts from local universities have taken the initiative to undergo training in genome editing techniques. Furthermore, Malawi collaborates with AUDA-NEPAD to spearhead a genome editing project, raising awareness about the benefits of genome editing in combating hunger and achieving the goals of Agenda 2063, the Africa We Want.

### 3.6 Mozambique

Mozambique’s government prioritizes agriculture in its Five-Year Program, using it as a guiding framework to shape national policy. The government makes this a top priority due to several compelling factors: nearly 70% of Mozambicans depend on farming for their livelihoods, and the sector generates around 26% of the country’s GDP. Given these significant statistics, any initiatives aimed at supporting and accelerating agricultural development are crucial for Mozambique, particularly amidst growing population pressures and the challenges posed by climate change (Arndt et al., 2010). Smallholder farms dominate Mozambique’s agricultural landscape, with commercial farming operations playing a relatively minor role. Unfortunately, the country’s agricultural productivity ranks among the lowest in the world (Nhantumbo et al., 2021). To address this issue, sustainable management of natural resources while simultaneously boosting agricultural production and productivity is essential. The National Strategy for Science, Technology, and Innovation (ENCTI) emphasizes that solid research must guide these efforts. By aligning agricultural development with scientific research and innovation, Mozambique can promote sustainable practices that not only increase output but also ensure long-term environmental preservation.

Researchers in Mozambique actively explore ways to enhance agricultural practices, applying various biotechnological tools and techniques to drive innovation. Initiatives aimed at boosting production, productivity, and addressing both biotic and abiotic stressors are particularly critical given the global advances in biotechnology that have transformed agricultural practices worldwide. Specifically, the application of biotechnology in Mozambique’s agricultural sector presents opportunities for improved yields, pest and disease resistance, and enhanced nutrient content in food crops. These advancements are vital for ensuring food security and improving livelihoods across the nation. Innovations in areas such as genetic engineering, precision farming, and soil microbiology hold immense potential for revolutionizing Mozambique’s agricultural landscape and contributing to the country’s broader sustainable development goals (Nhantumbo et al., 2021; Rooyen and Sigwele, 1998).

Significantly, across the African continent, substantial efforts are underway to adopt modern biotechnology, particularly genome editing. The Centre of Excellence in Science, Technology, and Innovation (CoE STI) established by AUDA-NEPAD is at the forefront of these initiatives. By building strategic partnerships, the center integrates innovations that bridge science and practical application, and spearheads a project to boost agricultural production in Africa. This initiative is part of broader efforts to harness the potential of modern biotechnology, driving industrial growth and fostering inclusive development to accelerate Africa’s socio-economic transformation (Thomson, 2007; Itam et al., 2023; Ochieng and Ananga, 2019).

Mozambique draws inspiration from Africa’s successful applications of modern biotechnology, particularly genome editing in agriculture, and actively participates in the CoE STI initiative led by AUDA-NEPAD. This initiative emphasizes leveraging genome editing technology to enhance staple food species, including maize, cassava, sorghum, wheat, yams, chickens, and cattle. Through this engagement, Mozambique aims to harness these technologies to improve its agricultural productivity and sustainability (AUDA-NEPAD, 2023; Ku and Ha, 2020). By participating in genome editing initiatives, Mozambique aspires to bolster national expertise among a diverse array of stakeholders, including policymakers, regulators, researchers, and the general public. Policymakers and educators are joining forces to build a skilled and informed workforce. Additionally, Mozambique plans to prioritize research and development focused on species where genome editing can facilitate tailored breeding programs to address their specific production needs.

Moreover, Mozambique perceives these continental and global initiatives as opportunities to enhance its research and development infrastructure and integrate itself into networks of modern biotechnology practitioners, particularly in genome editing. To further demonstrate its commitment to genome editing (GEd) technology, the country has developed and validated a Communication and Advocacy Strategy. The government implements this strategy to effectively communicate and advocate for genome editing technology among various stakeholders, and executes an action plan that outlines concrete steps and activities to enhance understanding and awareness.

Mozambique has made significant progress in adopting genome editing technology. The government has established guidelines for GEd use and approved a plan to edit specific genes in sorghum bicolor, enhancing light absorption and boosting crop density. However, despite these notable strides, there remains a need for strengthening regulatory frameworks and institutional capacity to ensure the responsible and effective deployment of genome editing and other biotechnologies within Mozambique’s agricultural sector (Ku and Ha, 2020; Entine et al., 2021).

## 4 SWOT analysis of genome editing in African agriculture

Amidst the pressing challenges of food insecurity, climate change, and rapid population growth, African nations are increasingly turning to innovative solutions to boost agricultural productivity. Biotechnology has yielded two prominent categories of products: GMOs and (GEd) products. As the distinction between GEd and GMOs becomes a critical point of debate, a comprehensive SWOT analysis is essential. This analysis dissects the strengths, weaknesses, opportunities, and threats associated with genome editing, providing a nuanced comparison with GMOs. By doing so, it informs evidence-based policy-making and agricultural strategies tailored to Africa’s unique challenges. Furthermore, this analysis enables a more effective assessment of the overall value and impact of these techniques (Gudanowska, 2014).

Genome editing boasts several distinctive advantages that underscore its potential to revolutionize agricultural innovation. One of the main advantages of genome editing is its precision, allowing scientists to make targeted changes at specific locations in the genome. This reduces the chance of unintended effects, making it a more appealing choice for improving specific traits. This precision also leads to greater efficiency, as genome editing speeds up the development of crop varieties. Researchers can create improved strains much faster than with traditional breeding methods or genetically modified approaches. When genome editing introduces changes similar to natural mutations, confined to endogenous traits, it mimics the natural process of mutation, yielding a comparable risk profile. This similarity underpins the rationale for distinct regulatory approaches, differing from those applied to GMOs. Consequently, genome editing often faces fewer regulatory hurdles, especially in regions like Africa, where it’s perceived as less intrusive (Wolt et al., 2016). This reduced regulatory burden, combined with its inherent biological similarities to natural mutations, can improve public acceptance and simplify approval processes, particularly when compared to transgenic GMOs (Enfissi et al., 2021).

This perspective helps hasten the development and commercialization of innovative crop varieties.

When evaluating genome editing, it is essential to weigh its numerous benefits against several notable weaknesses, particularly those that distinguish it from GMOs. One key limitation is the relatively limited understanding and experience with genome editing in agriculture. Although this technology has substantial potential, it is still emerging compared to the long-established history of GMOs. This newness may cause hesitance among stakeholders because of a lack of long-term data and potential challenges related to implementation. In addition, genome editing technologies tend to be technically complex and often require specialized tools and conditions that are not readily available in many African countries. Furthermore, intellectual property concerns regarding specific genome editing technologies can limit access, hindering their adoption in developing countries due to high costs or restrictive licensing agreements. To address these challenges, African governments should adopt a multi-faceted approach. This includes strengthening STEM education infrastructure and human capital, fostering international collaborations, and developing supportive policies for genome editing technologies. By doing so, research institutions will be empowered to conduct independent, Africa-based research, tackling food security issues. Additionally, establishing Technology Transfer Offices and Intellectual Property Offices within institutions will promote scientific innovation, protect scientists’ interests, and maximize the benefits of genome-edited products.

Realizing the potential of genome editing in African agriculture presents numerous opportunities for transformative impact. By enhancing crop yield and resilience, genome editing can play a vital role in boosting agricultural productivity. This technology enables the development of crop varieties that can withstand pests, diseases, and climatic stresses, significantly increasing food production and addressing food insecurity across the continent.

Moreover, countries like Nigeria, Malawi, Kenya, and Ghana are making significant strides by adopting a case-by-case regulatory approach to genome-edited products. This allows certain genome-edited varieties not to be classified as GMOs, thus streamlining the approval process and facilitating quicker access to innovative technologies. By promoting flexible regulatory environments, these countries can encourage research, development, and eventual commercialization of crop varieties tailored to the specific needs and challenges of local farmers.

Additionally, improved crop varieties can empower smallholder farmers by providing them with tools to increase profitability and reduce their vulnerability to climate fluctuations. This empowerment not only augments individual livelihoods but also stimulates economic growth within rural communities. By supporting smallholder farmers, genome editing can play a crucial role in achieving broader national and regional food security goals. Ultimately, embracing genome editing technologies fosters a collaborative environment, enabling African countries, researchers, and institutions to pool resources and knowledge. This cooperation enhances their ability to tackle common agricultural challenges more effectively and drives collective progress across the continent. By taking advantage of innovations in genome editing, African nations can position themselves at the forefront of agricultural technology, ensuring a sustainable and food-secure future for generations to come.

Genome editing’s potential is hindered by significant challenges, including consumer skepticism toward biotechnologies. Concerns about safety, ethics, and long-term impacts can impede acceptance of genome-edited crops, echoing the public trust issues faced by GMOs. In South Africa, the regulatory landscape poses a critical threat by categorizing genome-edited products under existing GMO regulations. This classification may intensify public scrutiny, influencing perceptions and regulatory attitudes. Consequently, South Africa’s regulatory stance on genome editing will have a ripple effect, serving as a potential blueprint for several African countries and shaping the continent’s approach to this technology. This influence extends to regional trade dynamics, consumer perceptions, and agricultural innovation. A fragmented market may emerge across Africa, with varying degrees of acceptance and regulation. Negative consumer reactions in South Africa could encourage skepticism in neighboring countries, heightening fears and resistance. Furthermore, ecological risks associated with genome-edited crops, such as crossbreeding affecting regional biodiversity, and transcend borders. Economically, South Africa’s regulatory decisions could impede biotechnology progress in agriculture across the continent, threatening potential advancements. While South Africa, as a sovereign state, has the right to make its own regulatory decisions, other African countries must also be empowered to develop and implement genome editing regulations that are tailored to their unique contexts and needs, based on independent and evidence-based decision-making. Regional bodies like the AU can facilitate this process by promoting regional cooperation and intensify information-sharing, and capacity-building on genome editing. This can include supporting country-led research initiatives, providing access to unbiased scientific expertise, and fostering inclusive dialogues that consider diverse perspectives. By adopting a collaborative and evidence-based approach, several African countries can harness the potential of genome editing while minimizing its risks and tailoring its applications to their specific development priorities.

## 5 Recommendations

Africa’s evolving agricultural technology landscape presents a crucial opportunity for researchers, policymakers, and stakeholders to glean actionable insights. To catalyze effective bio-innovation, particularly in the realm of genome editing, a holistic strategy is paramount. This necessitates a balanced evaluation encompassing innovation potential, socioeconomic implications, and the existing regulatory architecture, thereby establishing a robust foundation for sustainable agricultural practices. Our exhaustive analysis of Africa’s genome editing policy landscape, covering key countries such as Kenya, Nigeria, Malawi, Ghana, Mozambique, and Burkina Faso, yields pivotal recommendations to catalyze agricultural innovation via biotechnology.

Firstly, harmonizing policies and aligning national regulations with established continental frameworks, such as the CAADP, is very crucial. This strategic alignment fosters a cohesive regulatory environment, thereby promoting advancements in genome editing and facilitating broader technology adoption across the continent. To address existing knowledge deficits and mitigate technophobia surrounding genome editing, comprehensive education and outreach initiatives are indispensable. Implementing robust educational frameworks will empower diverse stakeholders by demystifying these technologies, facilitating informed dialogue, and promoting active engagement. This, in turn, creates a climate of trust and understanding. Regulatory flexibility is equally crucial. Advocating for adaptive regulatory frameworks that promote sustainable innovation will facilitate the rapid deployment and seamless integration of genome editing solutions into agricultural practices while ensuring adherence to stringent safety and sustainability standards.

Regional cooperation and collaborative partnerships among African nations are vital for the development of standardized policies tailored to unique agricultural contexts, ensuring consistency across borders. Effective monitoring mechanisms should be implemented to continuously evaluate the socioeconomic impacts of genome editing technologies. Strategic investments in capacity-building initiatives will enhance the comprehension and effective implementation of genome editing regulations among policymakers, scientists, and industry stakeholders. Prioritizing robust stakeholder engagement will foster continuous dialogue, address pertinent concerns, cultivate trust, and ensure that policies reflect diverse perspectives. Ultimately, a multidisciplinary approach that effectively integrates next-generation genome editing technologies is vital for addressing food insecurity and overcoming the multifaceted agricultural challenges confronting Africa. By strategically adopting these recommendations, African nations can effectively spearhead agricultural innovation, harnessing the transformative potential of genome editing technologies to secure a sustainable and food-secure future for the continent.
